# Integration vs Collaborative Redesign Strategies of Health Systems’ Supply Chains in the Post-COVID-19 New Normal: Cross-sectional Survey Across the United States

**DOI:** 10.2196/35317

**Published:** 2022-06-15

**Authors:** Jiban Khuntia, Frances J Mejia, Xue Ning, Jeff Helton, Rulon Stacey

**Affiliations:** 1 Health Administration Research Consortium University of Colorado Denver Denver, CO United States; 2 Business Department, University of Wisconsin Parkside Kenosha, WI United States

**Keywords:** COVID-19, post-COVID-19, health systems, supply chain integration, supply chain collaboration, supply chain resilience

## Abstract

**Background:**

Given the widespread disruptions to supply chains in 2020 because of the COVID-19 pandemic, questions such as how health systems are shaping strategies to restore the supply chain disruptions are essential to have confidence in health systems’ supply chain model strategies. Plausibly, health systems have an opportunity for redesign, growth, and innovation by utilizing collaborative strategies now, compared to the usual strategies of integrating their existing supply chains to reduce inefficiencies.

**Objective:**

This study focuses on teasing out the nuance of supply chain integration versus collaborative redesign strategies for health systems in the post-COVID-19 new normal. We focus on 2 research questions. First, we explore the impact of perceived supply chain challenges and disruptions on health systems’ supply chain integration (SC-INTEGRATION) and collaborative redesign (SC-REDESIGN) strategies. Second, we examine the outcomes of integration and collaborative redesign strategic choices on growth and service outcomes.

**Methods:**

We used data for this study collected through a consultant from a robust group of health system chief executive officers (CEOs) across the United States from February to March 2021. Among the 625 health system CEOs contacted, 135 (21.6%) responded to our survey. We considered supply chain–relevant strategy and outcome variables from the literature and ratified them via expert consensus. We collected secondary data from the Agency for Healthcare Research and Quality (AHRQ) Compendium of the US Health Systems, leading to a matched data set from the 124 health systems. Next, we used ordered logit model estimation to examine CEO preferences for partnership strategies to address current supply disruptions and the outcomes of strategy choices.

**Results:**

Health systems with higher disruptions would choose integration (positive, *P*<.001) over redesign, indicating that they still trust the existing partners. Integration strategy is perceived to result in better service outcomes (*P*<.01), while collaborations are perceived to lead to greater growth opportunities (*P*<.05); however, the role of integration in growth is not entirely ruled out (combined model, *P*<.001). Plausibly, some health systems would choose integration and collaborative redesign models, which have a significant relationship with both services (combined model, *P*<.01) and growth, establishing the importance of mixed strategies for health systems.

**Conclusions:**

The cost of health care continues to rise, and supply-related costs constitute a large portion of a hospital’s expenditure. Understanding supply chain strategic choices are essential for a health system’s success. Although collaboration is an option, focusing on and improving existing integration dynamics is helpful to foster both growth and services for health systems.

## Introduction

### Background

Supply chain disruptions during the COVID-19 pandemic presented multiple challenges for health systems. These included delays in several modes of transportation and delays in and inability to acquire critical supplies, leading to low inventories, backup suppliers sourcing from the same source as a critical supplier, and critical suppliers going into liquidation. A survey indicated that 10%-18% of health care organizations reported a significant impact, 4.1%-16% reported a severe impact, and 3%-5% reported a catastrophic impact on their businesses [1]. Supply chain disruptions adversely impacted operations, emergency plans, and responses during the pandemic and need to be revamped to improve health systems’ functions [2].

After the pandemic, health systems are in the process of restoring supply chains to be resilient. However, not all are blaming their current partners, as some of the challenges were beyond and above the scope of the supply chain delivery models—such as overly dependency on low-cost global value chains or lack of availability of alternative local vendors. Nevertheless, the disruptions invoked new efforts to at least focus on the supply chain resiliency and, accordingly, explore ways to focus on integration or redesign of supply chains along with other aspects [3]. It is essential to explore what factors influence health systems to choose between integration or redesign strategies and which of these strategies are effective for health system outcomes. The choice of either integration or collaboration strategies may be consequential or pose different challenges.

Prior research notes that uncooperative existing supply chain partners may lead to less cost-effective health outcomes or fail in implementing standardized health care processes [4,5]. In addition, redesigning a supply chain is not easy, as the complexities of supply chain management in the health care sector are high [4,6,7]. Nevertheless, since supply-related costs constitute 30%-50% of a hospital’s operating expenses [8], integrating or redesigning the supply chain is crucial for any health system.

The tension between adopting either integration or redesign is exacerbated postpandemic. Integration strategies adopted to cope with the supply chain challenges of the pandemic will require evaluation and modification to be sustainable over a longer-term, stable market landscape. Further, the compatibility of organizations that integrated during the pandemic should be evaluated to understand how sourcing strategies, pricing, and shipping logistics work on both supplier and user sides of the relationship [9,10].

Health systems appear to be involved in—or considering—integration with suppliers or redesigning supply chain relationships based on anecdotes and thorough responses to our survey. Thus, this study will focus on 2 research questions arising from these preliminary observations. First, we examine the impact of supply chain disruptions on health systems’ supply chain integration and redesign strategies. Second, we examine whether integration or redesign has a better outcome on growth and services.

Findings suggest that disruptions and challenges influence supply chain partnership choice. Higher disruptions tend to lead to integrative strategies. This finding could imply a greater trust among existing partners or the preference to avoid further burdening a complex system by adding more collaborative arrangements. Additionally, although collaboration provides greater growth opportunities, integration could also facilitate growth. The improvements in service delivery could provide resource allocation opportunities for organizations, allowing a shift in focus to growth initiatives.

Examining the supply chain strategies in health systems when facing challenges and the consequent outcomes will guide new supply chain management strategies and health care policies. Policy implementation to mitigate the financial risk associated with collaborative initiatives may impact partnership choice. Incentives to choose supply chain redesign despite its added complexity facilitate growth opportunities. Supply chain redesign through alternative sourcing facilities, for example, fosters resilience and provides sustainable solutions to supply chain disruptions.

### Literature Review: Integration or Redesign of Supply Chains

Operational challenges and supply chain inefficiencies have driven organizations to foster resilience through supply chain integration and redesign. The complexities of supply chain operations found within and between nodes contribute to supply chain disruptions. To integrate current systems to develop lean and agile supply chain operations, some organizations faced greater challenges due to the disruptions brought on by the COVID-19 pandemic. This has highlighted the importance of continual supply chain evaluations and the implementation of proactive strategies to foster resilience. This literature review examines the factors contributing to and the severity of supply chain disruptions. Next, we examine organizational resilience and how organizations have fostered resilience and have found supply chain sustainability solutions through supply chain integration and redesign.

Supply chain inefficiencies and disruptions present unique challenges for health systems. Factors specific to the health sector contribute to these challenges more. For instance, physicians are key decision makers in the procurement of prescription drugs but may have limited understanding of the production and supply chain or may think it is different from other sectors [4]. The health care sector is under regulatory pressures and long drug developmental cycles, and inventory management complexities in predicting a patient mix and supply consumption are added challenges [6]. Further, to maximize the benefit of supply chain operations, management must improve supplier relationship management, logistics operational tools, and process improvement, similar to other sectors.

The COVID-19 pandemic has exacerbated issues faced in supply chain operations. Some health care supply chains focus on minimizing costs by following a lean, agile inventory approach; however, during the pandemic, there was a pause in the movement of materials, causing massive supply chain disruptions. During the COVID-19 pandemic, health care providers’ personal protective equipment (PPE), medical-surgical supplies, and pharmaceuticals faced supply chain disruptions. Hospitals’ efforts to mitigate and minimize potential supply chain disruptions during COVID-19 were unsuccessful due to the overreliance on overseas manufacturing [11,12].

Health care organizations operate in disruptive environments. Continual evaluation of supply chain operations and proactive actions must be implemented to minimize critical supply shortages. Prior research points to developing and updating robust continuity of the supply chain through various means, such as an agile and innovative culture, communication practices, business continuity, and sourcing strategies [11-13]. However, in practice, a hospital may choose to engage in a spectrum of supply-related management, ranging from complete internal control to complete outsourcing. In this process, factors such as product or service characteristics, spatial complexity, degree of goal congruence, regulatory environment, and physical characteristics of the health system are vital to determine the strategic choices [6].

The processes and determinants of supply chain disruption severity include density, complexity, and node criticality [14]. Density is the quantity and geographic spacing of nodes in a supply chain; highly dense areas are more likely to experience significant disruptions if there is a significant portion of sourcing from those impacted areas. The complexity here refers to the relationship between the number of nodes in a supply chain and the number of connections among the nodes. On the one hand, a less complex system with fewer nodes and connections may experience less significant disruptions, but on the other hand, the presence of extra nodes can act as a buffer for supplier setbacks and can thus increase resiliency despite the added complexity. Node criticality is the relative importance of a node, and supply chains with a more significant number of critical nodes have greater probabilities of experiencing disruptions than supply chains in which critical processes are distributed or shared among several nodes. Supply chain integration drives optimal operations through the seamless exchange of information and the flow of products. Successful integration can increase organizational competitiveness through increased flexibility and responsiveness [15]. This change in operational strategy is standard and increases an organization’s capability to provide services.

The challenges of inefficient supply chains can drive organizations to foster resilience and overcome these challenges through supply chain integration and collaboration. Resilience can be defined as the ability of a supply chain system to reduce the probability of disruptions, reduce the consequences of disruptions, and reduce recovery time from disruptions back to normal operations [14]. Thus, supply chain resiliency starts with the organizations’ ability to proactively address unanticipated events. Suppliers can significantly impact the production of critical supplies used by health organizations, as seen by the COVID-19 pandemic [16].

Many organizations utilize information systems and integrate information technology into their supply chain operations to improve integration through infrastructural support, value creation, logistic operations, and supply chain management performance [16-18]. The utilization of information technology to optimize health care delivery includes telehealth, electronic medical records, and the implementation of decision support systems. Additionally, health care organizations have leveraged innovations by utilizing cloud-based information sharing to improve supply chain visibility and improve the system’s responsiveness to market forces [19]. Although associated privacy issues remain a significant concern in outsourcing health care data, privacy-preserving hybrid frameworks have been proposed to transit insensitive data to commercial cloud systems and the rest to trusted private cloud systems [20]; newer technologies, such as blockchains, provide several of the trusting and transparency-based tracking functions [21,22].

The use of cloud-based information sharing in hospitals is better positioned to efficiently respond to patient demand, reducing inventory costs, supply costs, and supply shortages. Radio-frequency identification (RFID) also improves inventory visibility at various supply chain stages to reduce shrinkage and shipping errors [5]. Supply chain redesign through start-ups or other entrepreneurial initiatives involves the collaboration of 2 or more organizations to execute supply chain operations, of which partnerships can be ongoing or limited.

Collaboration can be further divided into vertical and horizontal components. Vertical collaboration can include collaborations with customers, internal functions, and suppliers, while horizontal collaboration includes relationships with competitors and noncompetitors [23]. Supply chain collaboration is less chosen, possibly due to its difficulty to implement, overreliance on technology in trying to implement it, failure to differentiate between whom to collaborate with in the segmentation of customers and suppliers, a fundamental lack of trust between trading partners, and inequality between and among partners [23,24]. Despite collaboration challenges, these partnerships can foster organizational growth through innovation. Interconnecting elements in collaboration include information sharing, goal congruence, decision synchronization, incentive alignment, resource sharing, collaborative communication, and joint knowledge creation [25].

A successful collaboration leverages the knowledge and resources of suppliers and buyers. Large organizations may enter partnerships with start-ups because of the creativity, flexibility, and agility; large organizations can offer financial resources and execution of these ideas [26]. In 1 collaborative effort, physician leaders and stakeholder groups launched clinical communities focused on specialized care, including joint replacement, spine surgery, and blood management. The partnership between the supply chain team and lead physicians addressed the growing cost of health care through supply cost initiatives, realizing significant cost savings. The combined efforts of this initiative, which included medical supply consolidation, resulted in multimillion-dollar savings [27].

The current COVID-19 pandemic has brought to light weaknesses in the health care supply chain, resulting in delays or backorders of supply requisitions. The potential issues with delivery delays and backorders are myriad but include impacts such as duplicate orders or multiple orders to different vendors, resulting in excessive inventory holdings and poor working capital management. Cooperation between suppliers and buyers requires frequent communication on order status, shipping delays, coordination of substitute products as needed, and fair allocation of available stock among multiple buyers. Cooperation between these parties can also identify potential causes of backorders and shipping delays that ultimately improve the efficiency of product movement through the supply chain and optimize investments in inventory [28].

A common way to synthesize these multiple supply chain challenges is through a group purchasing organization (GPO), which represents a collaboration between suppliers, distributors, and end-user organizations to reduce costs and increase the efficiency of supply chain operations [8]. Although a GPO can reduce supply costs to health systems, it requires some compromises in standardizing item usage (eg, exam gloves or intravenous [IV] sets) to increase the bargaining power that yields such a low price. Many of the collaborative models noted earlier in this review have elements of cooperation between suppliers and buyers without the price negotiation dynamics. Nevertheless, successful integration and redesign strategies offer organizations service and growth opportunities. Supply chain integration has led to service improvements within organizations. The need for agility in supply chain operations has led organizations to leverage their integrated capabilities to create a more seamless exchange of information. Other organizations have leveraged collaborations to capitalize on business growth opportunities. Increased collaborative efforts by adding manufacturing nodes have allowed organizations to address shortage issues. Global supply chain sourcing was disrupted, facilitating the inclusion and collaboration of local sourcing facilities [29]. In this context, investigating the impact of supply chain disruptions on health systems’ supply chain integration and redesign strategies and whether integration or redesign has a better outcome on growth and services is timely and informative to both practice and research.

## Methods

### Data Collection

The effort to assess the linkage between the competition and integration prospects of health systems is part of a broad project undertaken by the Health Administration Research Consortium at the Business School of the University of Colorado Denver [30]. The project involves an annual and broad study on health systems and collects insights via a survey of health system chief executive officers (CEOs).

Data for this study were collected in collaboration with a consultant from a robust group of health system CEOs across the United States from January to March 2021. Expert inputs were taken, and the survey was validated and pilot-tested with 5 top executives from the Health Administration Program Advisory Board. The survey questionnaire was revised and finalized in January 2021. The specific questions that were asked in the survey instrument to measure the supply chain variables are as follows:

- I believe that the most pressing issue facing the growth of my health system in 2021 is supply chain disruptions and challenges.
- Are you currently engaged with or considering engaging with any of the following types of partners through joint ventures, strategic alliances, or informal collaborations to support your growth? The second question has the options of (1) supply chain and logistics organizations, (2) start-ups or entrepreneurial collaborations, (3) the new normal is presenting growth opportunities different than before COVID-19, (4) health delivery and services overall will improve over the next 12 months.

These questions used a 7-point Likert scale that varied from 1 = strongly disagree to 7 = strongly agree.

A contact list of CEOs was compiled from 624 health systems across the United States using data from multiple sources, contacts, professional networks, websites, and annual reports. The survey instrument was implemented in a professional survey platform and was mapped with emails to the platform to create unique, trackable links for each health system. Email and phone solicitations were made in multiple rounds between January 25 and March 2, 2021. A total of 148 responses were received from the 624 CEOs contacted, representing a 23.7% response rate, of which 13 (8.8%) incomplete responses could not be used, leaving 135 (21.6%) final usable responses. The 135 health systems represented in this survey varied from 1 to 18 hospitals with 176-75,000 employees. The annual revenue in 2020 of the health systems ranged from US $0.7 million to US $14 billion. The health systems aggregately represented US $300 billion in revenues and 1.1 million employees across the United States.

We then matched the survey data set with the secondary data collected from the Agency for Healthcare Research and Quality (AHRQ) *Compendium of the US Health Systems* to understand a better and complete picture of the health systems. Finally, we obtained data from 124 health systems located across the United States. We analyzed this combined data set to report several insights in this study.

### Ethics Consideration

An ethics review was not applicable for this study. The data used was received through a leading professional consulting firm that anonymizes and provides secondary firm-level data for research and analysis to draw insights.

### Variables and Measures

Table 1 shows the description of supply chain variables used in this study. The main variables in this study are supply chain disruptions and challenges (SC-DISRUP), presenting growth opportunities in the post-COVID-19 new normal (GROWTH), overall improvements in health delivery and services (SERVICE-IMPR), integration with supply chain and logistics organizations (SC-INTEGR), and redesign through start-ups or entrepreneurial collaborations (SC-REDEGN).

The influencing factors examined in this study are in several categories: size, region, teaching status, revenue, and several other system characteristics. These variables were coded in the way shown in Table 2 to reflect the characteristics of a health system, which may influence its supply chain disruptions, challenges, and growth in the post-COVID-19 new normal. In summary, 3 size variables measured the number of beds across a health system (SIZE_B-SMALL, SIZE_B-MEDIUM, SIZE_B-LARGE), 4 region variables reflected the location of a health system (REGION-NE, REGION-MW, REGION-SOUTH, REGION-WEST, where NE refers to “Northeast” and MW to “Midwest”), 3 variables were related to teaching status (TEACHING-NON, TEACHING-MINOR, TEACHING-MAJOR), and 3 revenue variables measured the annual revenue of a health system (REVENUE-LOW, REVENUE-MEDIUM, REVENUE-HIGH), in addition to variables about the disproportionate share hospital (DSH) patient (HIGH-DSH-HOSP), uncompensated care burden (HIGH-BURDEN-SYS and HIGH-BURDEN-HOSP), ownership status (OWNERSHIP), number of physicians (PHYSICIANS), and number of hospitals (HOSPITALS).

**Table 1:** Description of supply chain variables.

Variable	Description
SC-DISRUP^a^	Supply chain disruptions and challenges
SC-INTEGR^b^	Partner integration: supply chain and logistics organizations
SC-REDEGN^c^	Partner redesign: start-ups or entrepreneurial collaborations
GROWTH^d^	New normal: presenting growth opportunities
SERVICE-IMPR^e^	New normal: health delivery and services overall will improve

^a^SC-DISRUP: supply chain disruptions and challenges.

^b^SC-INTEGR: integration with supply chain and logistics organizations.

^c^SC-REDEGN: redesign through start-ups or entrepreneurial collaborations.

^d^GROWTH: presenting growth opportunities in the post-COVID-19 new normal.

^e^SERVICE-IMPR: overall improvements in health delivery and services.

**Table 2:** Description of contingent variables.

Variable		Description
**Size: The 3 size variables of the health system were coded using the total beds managed by the health system across all hospitals, reported by the AHRQ^a^ *Compendium of the US Health Systems*.**		
	SIZE_B-SMALL	The health system has <100 beds.
	SIZE_B-MEDIUM	The health system has 100-400 beds.
	SIZE_B-LARGE	The health system has >400 beds.
**Region: The 4 region variables of the health systems were coded based on their primary location in the United States, following the Census Bureau categorization.**		
	REGION-NE^b^	The health system in the Northeast
	REGION-MW^c^	The health system in Midwest
	REGION-SOUTH	The health system in the South
	REGION-WEST	The health system in the West
**Teaching: The 3 teaching variables were coded based on the teaching status of a health system.**		
	TEACHING-NON	A nonteaching health system
	TEACHING-MINOR	A minor teaching health system
	TEACHING-MAJOR	A major teaching health system
**Revenue: The 3 revenue variables of the health systems were coded using the annual revenue of the health system across all hospitals.**		
	REVENUE-LOW	Revenue<US $2 billion
	REVENUE-MEDIUM	Revenue=US $2-5 billion
	REVENUE-HIGH	Revenue>US $5 billion
HIGH-DSH^d^-HOSP		The health system includes at least 1 high-DSH-patient-percentage hospital: 1=yes, 0=no.
HIGH-BURDEN-SYS		Health system–wide uncompensated care burden flag: 1=yes, 0=no
HIGH-BURDEN-HOSP		The health system includes at least 1 high-uncompensated-care-burden hospital: 1=yes, 0=no.
OWNERSHIP		Predominantly investor-owned hospitals: 1=yes, 0=no
PHYSICIANS		The number of physicians in the health system is measured by the number of physicians reported by the AHRQ *Compendium of the US Health Systems*.
HOSPITALS		This is the number of hospitals the health system has, as reported by the AHRQ *Compendium of the US Health Systems*.

^a^AHRQ: Agency for Healthcare Research and Quality.

^b^NE: Northeast.

^c^MW: Midwest.

^d^DSH: disproportionate share hospital.

### Statistical Analysis

To answer the 2 research questions, we had 2 sets of analyses: (1) We used ordered logit regressions to estimate the direct relationships of the supply chain variables, future partner plans, and the outcomes, and (2) we used ordered logit regressions to estimate the mediation effects of the supply chain partner choices on outcomes. The variables were ordinal ones to drive the decision for ordered logit regressions. This approach does not assume equal intervals between levels in the dependent variable. The ordered logit model is as follows:

Y^*^_i_ = βX_i_ + e_i_,

where Y^*^_i_ is the propensity of respondents to indicate higher levels of the dependent variables (ie, SC-DISRUP, GROWTH, SERVICE-IMPR, SC-INTEGR, SC-REDEGN), X_i_ is a set of explanatory variables, *β* is a vector of parameters, and e_i_ are disturbances.

We did not observe Y^*^_i_. Instead, we observed the ordinal dependent variable Y_i_ depending on the values of thresholds or cut-off points *τ*_m–1_ and *τ*_m_, and the probability distribution of Y_i_ is given as follows:

Pr = [Y_i_ = m|X_i_ = F(τ_m_ – Xβ) – F(τ_m–1_ – Xβ)]

## Results

### Sample Statistics

The descriptive statistics and pairwise correlations among the key variables used in this study are presented in Tables 3 and 4. As shown in Table 3, health systems face relatively high supply chain disruptions and challenges (SC-DISRUP mean 4.45, SD 1.82). Additionally, the anticipation of improving health delivery and services overall (SERVICE-IMPR mean 3.48, SD 2.07) seemed to be a more popular outlook among CEOs over the presentation of growth opportunities (GROWTH mean 3.08, SD 2.01). The partnership choice of integrating with supply chain and logistics organizations (SC-INTEGR mean 3.98, SD 2.15) is similar to redesign through start-ups or entrepreneurial collaborations (SC-REDEGN mean 3.97, SD 2.15). Table 4 presents the correlations between the main variables.

**Table 3:** Summary statistics (N=124).

Variable	Mean (SD)	Min.	Max.
SC-DISRUP^a^	4.45 (1.82)	1	7
GROWTH^b^	3.08 (2.01)	1	7
SERVICE-IMPR^c^	3.48 (2.07)	1	7
SC-INTEGR^d^	3.98 (2.15)	1	7
SC-REDEGN^e^	3.97 (2.15)	1	7
SIZE_B-SMALL	0.08 (0.27)	0	1
SIZE_B-MEDIUM	0.40 (0.49)	0	1
SIZE_B-LARGE	0.52 (0.50)	0	1
REGION-NE^f^	0.22 (0.42)	0	1
REGION-MW^g^	0.26 (0.44)	0	1
REGION-SOUTH	0.34 (0.47)	0	1
REGION-WEST	0.18 (0.38)	0	1
TEACHING-NON	0.30 (0.46)	0	1
TEACHING-MINOR	0.44 (0.50)	0	1
TEACHING-MAJOR	0.26 (0.44)	0	1
REVENUE-LOW	0.12 (0.33)	0	1
REVENUE-MEDIUM	0.05 (0.22)	0	1
REVENUE-HIGH	0.83 (0.38)	0	1
HIGH-DSH^h^-HOSP	0.33 (0.47)	0	1
HIGH-BURDEN-SYS	0.19 (0.39)	0	1
HIGH-BURDEN-HOSP	0.32 (0.47)	0	1
OWNERSHIP	0.03 (0.16)	0	1
PHYSICIANS	1.86 (0.77)	1	3
HOSPITALS	1.50 (0.81)	1	3

^a^SC-DISRUP: supply chain disruptions and challenges.

^b^GROWTH: presenting growth opportunities in the post-COVID-19 new normal.

^c^SERVICE-IMPR: overall improvements in health delivery and services.

^d^SC-INTEGR: integration with supply chain and logistics organizations.

^e^SC-REDEGN: redesign through start-ups or entrepreneurial collaborations.

^f^NE: Northeast.

^g^MW: Midwest.

^h^DSH: disproportionate share hospital.

**Table 4:** Pairwise correlations between main variables (N=124).

		1	2	3	4	5	6	7	8	9	10	11	12	13	14	15	16	17	18	19	20
1	SC-DISRUP^a^	1.00	-.03	.25	.69	-.46	.02	-.04	.14	-.23	-.01	.07	-.06	.08	-.13	.06	-.11	.04	-.08	-.01	.02
2	GROWTH^b^	–.03	1.00	.26	.07	.17	-.14	.22	-.06	.04	-.01	-.10	.03	.03	-.01	.04	-.01	.06	-.12	.16	.13
3	SERVICE-IMPR^c^	.25	.26	1.00	.20	-.04	.04	.06	.10	-.07	.03	-.12	-.03	.08	-.11	.10	-.06	.13	.07	.10	.02
4	SC-INTEGR^d^	.69	.07	.20	1.00	-.23	.03	-.08	.03	-.11	-.01	.04	-.04	-.22	-.25	-.08	-.07	.10	-.11	-.15	-.05
5	SC-REDEGN^e^	–.46	.17	–.04	–.23	1.00	-.05	.09	-.02	.17	.12	-.15	-.06	.08	.15	-.17	.05	-.05	.01	-.01	.00
6	SIZE_B-MED	.02	–.14	.04	.03	–.05	1.00	-.83	-.08	-.01	-.05	.03	-.10	-.16	-.27	-.32	-.08	-.01	-.21	-.60	-.46
7	SIZE_B-LARGE	–.04	.22	.06	–.08	.09	–.83	1.00	-.01	.11	.01	-.14	.26	.25	.36	.38	.17	.06	.28	.75	.54
8	REGION-MW^f^	.14	–.06	.10	.03	–.02	–.08	–.01	1.00	-.42	-.26	-.07	-.01	-.12	-.09	.09	-.20	-.10	.00	.02	.02
9	REGION-SOUTH	–.23	.04	–.07	–.11	.17	–.01	.11	–.42	1.00	-.34	.17	.07	.02	.15	-.02	.12	.17	.22	.00	.04
10	REGION-WEST	–.01	–.01	.03	–.01	.12	–.05	.005	–.26	–.34	1.00	-.06	-.02	-.04	-.06	-.01	.12	.08	.02	-.04	-.03
11	TEACHING-MINOR	.07	–.10	–.12	.04	–.15	.03	–.14	–.07	.17	–.06	1.00	.01	-.07	-.07	-.05	.05	-.06	-.08	-.05	-.08
12	TEACHING-MAJOR	–.06	.03	–.03	–.04	–.06	–.10	.26	–.01	.07	–.02	.01	1.00	-.50	.12	.07	.05	-.12	.08	.19	.26
13	REVENUE-MEDIUM	–.08	.03	.08	–.22	.08	–.16	.25	–.12	.02	–.04	–.07	–.50	1.00	.12	.17	.34	.03	.13	.38	.06
14	REVENUE-HIGH	–.13	–.01	–.11	–.25	.15	–.27	.36	–.09	.15	–.06	–.07	.12	.12	1.00	-.23	-.06	-.04	.14	.26	.29
15	HIGH-DSH^g^-HOSP	.06	.04	.10	–.08	–.17	–.32	.38	.09	–.02	–.01	–.05	.07	.17	–.23	1.00	.15	-.04	-.02	.51	.30
16	HIGH-BURDEN-SYS	–.11	–.01	–.06	–.07	.05	–.08	.17	–.20	.12	.12	.05	.05	.34	–.06	.15	1.00	-.01	.18	.23	.17
17	HIGH-BURDEN-HOSP	.04	.06	.13	.10	–.05	–.01	.06	–.10	.17	.08	–.06	–.12	.03	–.04	–.04	–.01	1.00	.42	-.10	-.20
18	OWNERSHIP	–.08	–.12	.07	–.11	.01	–.21	.28	.002	.22	.02	–.08	.08	.13	.14	–.02	.18	.42	1.00	.18	.31
19	PHYSICIANS	–.01	.16	.10	–.15	–.01	–.60	.75	.02	.002	–.04	–.05	.19	.38	.26	.51	.23	–.10	.18	1.00	.57
20	HOSPITALS	.02	.13	.02	–.05	.001	–.46	.54	.02	.04	–.03	–.08	.26	.06	.29	.30	.17	–.20	.31	.57	1.00

^a^SC-DISRUP: supply chain disruptions and challenges.

^b^GROWTH: presenting growth opportunities in the post-COVID-19 new normal.

^c^SERVICE-IMPR: overall improvements in health delivery and services.

^d^SC-INTEGR: integration with supply chain and logistics organizations.

^e^SC-REDEGN: redesign through start-ups or entrepreneurial collaborations.

^f^MW: Midwest.

^g^DSH: disproportionate share hospital.

### Estimation Outcomes

Table 5 shows the first set of results of the ordered logit model estimation that describes the relationship between perception of supply chain–related challenges (SC-DISRUP) and future partnership plans: either integrating with supply chain and logistics organizations (SC-INTEGR) or redesigning through start-ups or entrepreneurial collaborations (SC-REDEGN).

We found a positive and significant relationship between the supply chain issue and supply chain partner choice. With a higher expectation of supply chain disruption and challenges, health systems partner with supply chain and logistics organizations and integrate it better. In addition, there was a negative and significant relationship between supply chain issue and start-up partner choice, indicating that with higher expectations of supply chain disruption and challenges, health systems tend *not* to partner with start-ups or entrepreneurial collaborations (ie, redesigning supply chain using new start-ups).

Table 6 shows the direct relationships between supply chain partnership plans and outcomes. First, there are positive and significant relationships between supply chain partner and service in the separate models and between start-up partner and growth. Both partner options can contribute to the growth in the combined models, while supply chain partners can significantly increase service improvement. In other words, integration is better for service and growth, but redesign is better for growth. The results indicate that with solid supply chain support by integration, health systems can provide better services; with redesign, health systems can increase the innovation/capability needed for growth.

We coded a new variable to capture such dual options to explore the impacts of supply chain partnership plans when health systems have integration and redesign plans. This variable is a dummy variable that equals 1 if both SC-INTEGR and SC-REDEGN are greater than their mean values, or 0 otherwise. Table 7 shows the direct relationships between supply chain challenges and dual-partnership (DUAL) plans and between dual-partnership plans and outcomes. We found no significant relationship between perceived supply chain challenges and dual-partnership choices. There are significant relationships between dual-partnership choices and growth and service outcomes, indicating that health systems can increase growth and service improvement when health systems choose both partner options.

After examining the direct relationships, we tested the mediating effects of supply chain partnership plans on the outcomes. Table 8 displays the impacts on growth, and Table 9 presents the impacts on service. We found that supply chain challenges do not directly affect growth and service outcomes, and partnership plans can mediate these relationships. We further conducted the Sobel mediation test to check the mediation effects. We found that supply chain integration partner choice has a significant and positive impact on growth outcome; the mediating effect is 0.84 (proportion of the total effect that is mediated). In addition, the redesign through start-up choice has a significant and positive impact on growth outcome (DV), and the mediating effect is 0.75. In contrast, for the impacts on service outcome, we found that the mediating effect of supply chain integration partner choice is only 0.18, while the mediating effect of redesign through start-up choice is only 0.08.

**Table 5:** Direct relationship between supply chain perceived challenges and partnership plans.^a^

Variables	SC-INTEGR^b,c^		SC-REDEGN^d,e^	
	Coefficient (SE)	*P* value	Coefficient (SE)	*P* value
SC-DISRUP^f^	.858 (0.049)	<.001	–.478 (0.058)	<.001
SIZE	.599 (0.486)	.22	.693 (0.060)	<.001
REGION	–.129 (0.217)	.55	.466 (0.133)	<.001
OWNERSHIP	–.522 (0.598)	.38	–1.541 (0.534)	.004
TEACHING	–.962 (0.110)	<.001	–.122 (0.490)	.80
REVENUE	–.826 (0.270)	.002	–.479 (0.494)	.33
HIGH-DSH^g^-HOSP	.839 (0.489)	.09	–.025 (0.176)	.89
HIGH-BURDEN-SYS	.400 (0.301)	.18	–.547 (0.069)	<.001
HIGH-BURDEN-HOSP	–.257 (0.092)	.01	–.374 (0.231)	.11
PHYSICIANS	–.016 (0.444)	.97	–.082 (0.112)	.47
HOSPITALS	.268 (0.144)	.06	.018 (0.182)	.92

^a^The results of the cut-off points are omitted for brevity.

^b^SC-INTEGR: integration with supply chain and logistics organizations.

^c^Pseudo *R^2^*=.2038 (n=123 observations).

^d^SC-REDEGN: redesign through start-ups or entrepreneurial collaborations.

^e^Pseudo *R^2^*=.1112 (n=124 observations).

^f^SC-DISRUP: supply chain disruptions and challenges.

^g^DSH: disproportionate share hospital.

**Table 6:** Direct relationship between supply chain partnership plans and outcomes.^a^

Variables	GROWTH^b,c^		SERVICE-IMPR^d,e^		GROWTH^f^		SERVICE-IMPR^g^		GROWTH^h^		SERVICE-IMPR^i^	
	Coefficient (SE)	*P* value	Coefficient (SE)	*P* value	Coefficient (SE)	*P* value	Coefficient (SE)	*P* value	Coefficient (SE)	*P* value	Coefficient (SE)	*P* value
SC-INTEGR^j^	.096 (0.060)	.11	.327 (0.117)	.005	N/A^k^	N/A	N/A	N/A	.142 (0.038)	*P*<.001	.335 (0.120)	.01
SC-REDEGN^l^	N/A	N/A	N/A	N/A	.200 (0.086)	.02	–.010 (0.125)	.94	.212 (0.083)	.01	.037 (0.140)	.79
SIZE	.915 (0.362)	.01	.165 (0.597)	.78	.852 (0.380)	.03	.319 (0.472)	.50	.793 (0.377)	.04	.145 (0.512)	.78
REGION	.053 (0.173)	.76	.182 (0.066)	.006	–.116 (0.207)	.58	.078 (0.102)	.45	–.076 (0.256)	.77	.165 (0.121)	.17
OWNERSHIP	–.792 (0.922)	.39	–1.484 (0.766)	.05	–.221 (0.642)	.73	–1.298 (0.377)	.001	–.210 (0.724)	.77	–1.378 (0.490)	.01
TEACHING	–.187 (0.133)	.16	.295 (0.036)	<.001	–.199 (0.030)	<.001	.082 (0.062)	.19	–.151 (0.008)	<.001	.293 (0.017)	<.001
REVENUE	–.762 (0.199)	<.001	–.133 (0.237)	.58	–.727 (0.246)	.003	–.320 (0.176)	.07	–.622 (0.265)	.02	–.117 (0.254)	.65
HIGH-DSH^m^-HOSP	–.207 (0.174)	.23	–.461 (0.266)	.08	–.124 (0.072)	.09	–.345 (0.173)	.05	–.204 (0.137)	.14	–.460 (0.261)	.08
HIGH-BURDEN-SYS	.867 (0.289)	.003	.313 (0.273)	.25	1.119 (0.396)	.01	.525 (0.142)	<.001	1.055 (0.305)	.001	.326 (0.239)	.17
HIGH-BURDEN-HOSP	–1.363 (0.229)	<.001	–.098 (0.169)	.56	–1.457 (0.116)	<.001	–.172 (0.100)	.09	–1.415 (0.142)	<.001	–.092 (0.146)	.53
PHYSICIANS	.312 (0.080)	<.001	.270 (0.218)	.22	.291 (0.125)	.02	.257 (0.157)	.10	.314 (0.165)	.06	.276 (0.196)	.16
HOSPITALS	.442 (0.055)	<.001	–.149 (0.096)	.12	.532 (0.039)	<.001	–.035 (0.100)	.73	.460 (0.067)	<.001	–.146 (0.085)	.08

^a^The results of the cut-off points are omitted for brevity.

^b^GROWTH: presenting growth opportunities in the post-COVID-19 new normal.

^c^Pseudo *R^2^*=.0583 (N= 123 observations).

^d^SERVICE-IMPR: overall improvements in health delivery and services.

^e^Pseudo *R^2^*=.0347 (N= 123 observations).

^f^Pseudo *R^2^*=.0689 (N= 124 observations).

^g^Pseudo *R^2^*=.0162 (N= 124 observations).

^h^Pseudo *R^2^*=.0717 (N= 123 observations).

^i^Pseudo *R^2^*=.0350 (N= 123 observations).

^j^SC-INTEGR: integration with supply chain and logistics organizations.

^k^N/A: not applicable.

^l^SC-REDEGN: redesign through start-ups or entrepreneurial collaborations.

^m^DSH: disproportionate share hospital.

**Table 7:** Direct relationship between supply chain challenges, dual-partnership plans, and outcomes.^a^

Variables	DUAL^b,c^		GROWTH^d,e^		SERVICE-IMPR^f,g^	
	Coefficient (SE)	*P* value	Coefficient (SE)	*P* value	Coefficient (SE)	*P* value
SC-DISRUP^h^	.035 (0.135)	.796	N/A^i^	<.001	N/A	N/A
DUAL	N/A	N/A	.848 (0.053)	<.001	.696 (0.354)	.05
SIZE	.526 (0.169)	.002	.916 (0.299)	.002	.254 (0.408)	.53
REGION	.293 (0.302)	.33	–.058 (0.184)	.75	.056 (0.164)	.74
OWNERSHIP	–14.273 (1.144)	<.001	–.298 (0.747)	.69	–.920 (1.297)	.48
TEACHING	–.294 (0.763)	.70	–.167 (0.011)	<.001	.133 (0.308)	.67
REVENUE	–1.150 (0.095)	<.001	–.648 (0.201)	.001	–.188 (0.305)	.54
HIGH-DSH^j^-HOSP	–.179 (0.384)	.64	–.069 (0.146)	.64	–.323 (0.375)	.39
HIGH-BURDEN-SYS	.590 (0.220)	.01	.921 (0.365)	.01	.429 (0.510)	.40
HIGH-BURDEN-HOSP	–.633 (0.149)	<.001	–1.408 (0.185)		–.145 (0.443)	.74
PHYSICIANS	.370 (0.572)	.52	.158 (0.117)	.18	.210 (0.342)	.54
HOSPITALS	.247 (0.151)	.10	.537 (0.066)	<.001	–.051 (0.276)	.85

^a^The results of the cut-off points are omitted for brevity.

^b^DUAL: dual partnership.

^c^Pseudo *R^2^*=.1067 (N=124 observations).

^d^GROWTH: presenting growth opportunities in the post-COVID-19 new normal.

^e^Pseudo *R^2^*=.0703 (N=124 observations).

^f^SERVICE-IMPR: overall improvements in health delivery and services.

^g^Pseudo *R^2^*=.0257 (N=124 observations).

^h^SC-DISRUP: supply chain disruptions and challenges.

^i^N/A: not applicable.

^j^DSH: disproportionate share hospital.

**Table 8:** Mediating effects of supply chain partnership plans on growth outcomes.^a^

Variables	GROWTH^b,c^		GROWTH^d^		GROWTH^e^		GROWTH^f^	
	Coefficient (SE)	*P* value	Coefficient (SE)	*P* value	Coefficient (SE)	*P* value	Coefficient (SE)	*P* value
SC-DISRUP^g^	–.064 (0.053)	.23	–.170 (0.073)	.02	.028 (0.023)	.22	–.068 (0.061)	.27
SC-INTEGR^h^	N/A^i^	N/A	.224 (0.048)	<.001	N/A	N/A	.189 (0.065)	.004
SC-REDEGN^j^	N/A	N/A	N/A	N/A	.209 (0.085)	.01	.195 (0.085)	.02
SIZE	.954 (0.374)	.01	.873 (0.391)	.03	.848 (0.372)	.02	.787 (0.389)	.04
REGION	–.006 (0.134)	.96	.028 (0.179)	.88	–.116 (0.210)	.58	–.073 (0.256)	.78
OWNERSHIP	–.702 (0.787)	.37	–.616 (0.883)	.49	–.226 (0.640)	.72	–.186 (0.725)	.80
TEACHING	–.230 (0.129)	.08	–.181 (0.116)	.12	–.187 (0.018)	<.001	–.153 (0.011)	<.001
REVENUE	–.846 (0.172)	<.001	–.717 (0.163)	<.001	–.720 (0.249)	.004	–.614 (0.249)	.01
HIGH-DSH^k^-HOSP	–.135 (0.107)	.21	–.250 (0.175)	.15	–.123 (0.075)	.10	–.224 (0.153)	.14
HIGH-BURDEN-SYS	.950 (0.405)	.02	.889 (0.324)	.01	1.114 (0.384)	.004	1.045 (0.312)	.001
HIGH-BURDEN-HOSP	–1.394 (0.165)	<.001	–1.340 (0.186)	<.001	–1.454 (0.124)	<.001	–1.400 (0.141)	<.001
PHYSICIANS	.301 (0.064)	<.001	.354 (0.124)	.004	.280 (0.127)	.03	.332 (0.192)	.09
HOSPITALS	.522 (0.062)	<.001	.465 (0.051)	<.001	.527 (0.041)	<.001	.467(0.046)	<.001

^a^The results of the cut-off points are omitted for brevity.

^b^GROWTH: presenting growth opportunities in the post-COVID-19 new normal.

^c^Pseudo *R^2^*=.0578 (N=124 observations).

^d^Pseudo *R^2^*=.0625 (N=123 observations).

^e^Pseudo *R^2^*=.0691 (N=124 observations).

^f^Pseudo *R^2^*=.0723 (N=123 observations).

^g^SC-DISRUP: supply chain disruptions and challenges.

^h^SC-INTEGR: integration with supply chain and logistics organizations.

^i^N/A: not applicable.

^j^SC-REDEGN: redesign through start-ups or entrepreneurial collaborations.

^k^DSH: disproportionate share hospital.

**Table 9:** Mediating effects of supply chain partnership plans on service outcomes.^a^

Variables	SERVICE-IMPR^b,c^		SERVICE-IMPR^d^		SERVICE-IMPR^e^		SERVICE-IMPR^f^	
	Coefficient (SE)	*P* value	Coefficient (SE)	*P* value	Coefficient (SE)	*P* value	Coefficient (SE)	*P* value
SC-DISRUP^g^	.289 (0.113)	.01	.201 (0.101)	.05	.310 (0.089)	.001	.221 (0.060)	<.001
SC-INTEGR^h^	N/A^i^	N/A	.178 (0.130)	.17	N/A	N/A	.177 (0.133)	.18
SC-REDEGN^j^	N/A	N/A	N/A	N/A	.073 (0.135)	.59	.071 (0.136)	.60
SIZE	.303 (0.544)	.58	.216 (0.563)	.70	.261 (0.454)	.57	.179 (0.478)	.71
REGION	.143 (0.047)	.002	.197 (0.059)	.001	.109 (0.094)	.25	.164 (0.119)	.17
OWNERSHIP	–1.680 (0.637)	.01	–1.684 (0.690)	.02	–1.489 (0.461)	.001	–1.498 (0.499)	.003
TEACHING	.221 (0.041)	<.001	.274 (0.034)	<.001	.219 (0.026)	<.001	.271 (0.017)	<.001
REVENUE	–.293 (0.262)	.26	–.179 (0.292)	.54	–.265 (0.284)	.35	–.153 (0.323)	.64
HIGH-DSH^k^-HOSP	–.331 (0.195)	.09	–.416 (0.259)	.11	–.330 (0.193)	.09	–.414 (0.255)	.10
HIGH-BURDEN-SYS	.321 (0.258)	.21	.268 (0.328)	.41	.353 (0.233)	.13	.295 (0.315)	.35
HIGH-BURDEN-HOSP	–.038 (0.236)	.87	–.056 (0.237)	.81	–.025 (0.207)	.90	–.042 (0.209)	.84
PHYSICIANS	.185 (0.221)	.40	.240 (0.206)	.24	.194 (0.196)	.32	.248 (0.186)	.18
HOSPITALS	–.120 (0.109)	.27	–.188 (0.112)	.09	–.115 (0.107)	.28	–.185 (0.115)	.11

^a^The results of the cut-off points are omitted for brevity.

^b^SERVICE-IMPR: overall improvements in health delivery and services.

^c^Pseudo *R^2^*=.0353 (N=124 observations).

^d^Pseudo *R^2^*=.0403 (N=123 observations).

^e^Pseudo *R^2^*=.0365 (N=124 observations).

^f^Pseudo *R^2^*=.0414 (N=123 observations).

^g^SC-DISRUP: supply chain disruptions and challenges.

^h^SC-INTEGR: integration with supply chain and logistics organizations.

^i^N/A: not applicable.

^j^SC-REDEGN: redesign through start-ups or entrepreneurial collaborations.

^k^DSH: disproportionate share hospital.

## Discussion

### Principal Findings

This study first explores the relationship between the perceived severity of supply chain–related challenges/disruptions and an organization’s future partnership plans to address those challenges. Second, the study elucidates the relationship between supply chain partnerships and outcomes. We also explored the relationship between supply chain challenges and a dual-partnership mix.

First, the results indicate that organizations tend to choose supply chain integration with existing partners as an operational strategy with higher perceived challenges and disruptions. These organizations tend not to partner with start-ups or other new entities.

Health systems with HIGH-DSH-HOSP and HIGH-BURDEN-SYS both tend to choose integration over collaboration. REGION and REVENUE negatively correlate with integration and positively correlate with collaboration. OWNERSHIP, TEACHING hospitals, HIGH-BURDEN-HOSP, and PHYSICIANS negatively correlate with integration and collaboration. SIZE and HOSPITALS both have a positive correlation with integration and collaboration.

Second, the results indicate a significant relationship between supply chain integration and service improvement and between supply chain collaborations and growth. Together, integration and collaboration contribute to growth, while integration has a more profound positive impact on service improvement. Thus, integration is better for both service and growth, while collaboration is better for growth.

There is a positive correlation with both GROWTH and SERVICE-IMPR for SIZE, REGION, HIGH-BURDEN-SYS, and PHYSICIANS in health systems that choose to integrate with existing partners. OWNERSHIP, REVENUE, HIGH-DSH-HOSP, and HIGH-BURDEN-HOSP negatively correlate with GROWTH and SERVICE-IMPR. TEACHING hospitals negatively correlate with GROWTH and have a positive correlation with SERVICE-IMPR. Thus, hospitals have a positive correlation with GROWTH and a negative correlation with SERVICE-IMPR.

For health systems that choose supply chain redesign, there is a positive correlation with both GROWTH and SERVICE-IMPR for the variables SIZE, HIGH-BURDEN-SYS, and PHYSICIANS. There is a negative correlation with GROWTH and a positive correlation with SERVICE-IMPR for REGION and TEACHING. There is a negative correlation with both GROWTH and SERVICE-IMPR for OWNERSHIP, REVENUE, HIGH-DSH-HOSP, and HIGH-BURDEN-HOSP. Thus, hospitals have a positive correlation with GROWTH and a negative correlation with SERVICE-IMPR.

Third, the results indicate no significant relationship between supply chain challenges and dual-partnership choice; however, dual-partnership choices significantly affected both service and growth. Taken together, the results suggest that health systems are better able to provide services with supply chain integration. Health systems positioned for growth can also increase innovation and capability through supply chain redesign.

For health systems that have dual partnerships, there is a positive correlation with both GROWTH and SERVICE-IMPR for the variables SIZE, HIGH-BURDEN-SYS, and PHYSICIANS. There is a negative correlation with GROWTH and a positive correlation with SERVICE-IMPR for REGION and TEACHING. There is a negative correlation with GROWTH and SERVICE-IMPR for OWNERSHIP, REVENUE, HIGH-DSH-HOSP, and HIGH-BURDEN-HOSP. Thus, hospitals have a positive correlation with GROWTH and a negative correlation with SERVICE-IMPR.

### Implications

The first set of results sheds light on the influence of perceived disruptions and challenges on partnership choice. Higher expectations of supply chain disruptions tend to influence supply chains to integrate more with existing partners and not with start-ups or other entrepreneurial collaborations. A plausible explanation is that high-burden systems may not want to pursue new collaborations to further add to the complexity of their current state. Additionally, health systems with high revenue may opt for supply chain redesign because of the financial resources available for the collaborative initiative.

The second set of findings investigates partnership choice and outcomes. Supply chain integration leads to greater service outcomes, while collaborations lead to greater growth opportunities. Although integration shows a more profound impact on service improvement, there are growth opportunities within this partnership choice. One possible explanation for these findings is that large high-burden systems have greater opportunity for growth within the supply chain when operations become more integrated and the flow of resources becomes more seamless. Resources can be redirected to growth initiatives.

Examining the factors that lead organizations to integration or collaboration will guide new strategies and policies in health care. Policy implementations that can minimize the financial risks of collaborations may drive dual-partnership choices. Healthy systems and health systems that do not perceive many challenges have an opportunity for competitive advantage through start-up collaborations. Additionally, organizations may contract with alternative suppliers for emergencies to ensure supply procurement in events that halt shipments. Future research could elucidate the impact of highly networked systems on perceived supply chain disruptions and thus partnership choice.

### Limitations and Directions for Future Research

The authors acknowledge some limitations of this study that future research could address. Integration and collaboration may not lead to increased service outcomes to growth opportunities if they are not implemented successfully, so providers, members of the supply chain team, and policymakers should focus on the conditions with which integration and collaboration can be successfully implemented to deliver greater value in the care continuum.

First, research could further examine other variables affecting partnership choice. This study focused on the perception of supply chain disruptions and challenges. However, other external and internal disruptions could affect partnership choice. Demand-driven externalities could also influence partnership choice.

Second, supply chain operations can be highly networked or have few key players. In health systems with few suppliers, an impact on a critical supplier will cause more significant disruptions in production. Organizations may collaborate with other suppliers in this case because further integration in the supply chain may not increase supply procurement.

### Conclusion

Disruptions in health care remain a challenge, partly due to supply chain management inefficiencies. Understanding these inefficiencies and how they impact supply chain partnership choice as an organizational strategy is essential in the health care sector. This study begins to investigate supply chain management partnership dynamics.

Health care organizations continuously face growing costs and meet these challenges through seamless integration efforts and innovative collaborations. Although higher perceptions of supply chain challenges lead to integration strategies rather than innovative redesign, integration and collaboration can provide lean and agile systems and better equip organizations to address challenges. Organizations have an opportunity to provide better service outcomes while also identifying and capitalizing on innovative growth opportunities.

## Abbreviations

AHRQ: Agency for Healthcare Research and Quality
CEO: chief executive officer
DSH: disproportionate share hospital
DUAL: dual partnership
GPO: group purchasing organization
GROWTH: presenting growth opportunities in the post-COVID-19 new normal
MW: Midwest
NE: Northeast
PPE: personal protective equipment
SC-DISRUP: supply chain disruptions and challenges
SC-INTEGR: integration with supply chain and logistics organizations
SC-REDEGN: redesign through start-ups or entrepreneurial collaborations
SERVICE-IMPR: overall improvements in health delivery and services

## References

[1] Mazareanu E Impact of the Coronavirus Pandemic on Supply Chains across Industries 2020.

[2] Guest JL, Del Rio C, Sanchez T (2020). The three steps needed to end the COVID-19 pandemic: bold public health leadership, rapid innovations, and courageous political will. JMIR Public Health Surveill.

[3] Khuntia J, Ning X, Stacey R (2022). Competition and integration of US health systems in the post-COVID-19 new normal: cross-sectional survey. JMIR Form Res.

[4] Kwon IG, Kim S, Martin DG (2016). Healthcare supply chain management; strategic areas for quality and financial improvement. Technol Forecast Soc Change.

[5] Mathew J, John J, Kumar S, Management O (2013). New trends in healthcare supply chain.

[6] Bhakoo V, Singh P, Sohal A (2012). Collaborative management of inventory in Australian hospital supply chains: practices and issues. Supply Chain Manag.

[7] Aronsson H, Abrahamsson M, Spens K (2011). Developing lean and agile health care supply chains. Supply Chain Manag.

[8] Francis JR (2020). COVID-19: implications for supply chain management. Front Health Serv Manag.

[9] Iyengar KP, Vaishya R, Bahl S, Vaish A (2020). Impact of the coronavirus pandemic on the supply chain in healthcare. Br J Healthc Manag.

[10] Nikolopoulos K, Punia S, Schäfers A, Tsinopoulos C, Vasilakis C (2021). Forecasting and planning during a pandemic: COVID-19 growth rates, supply chain disruptions, and governmental decisions. Eur J Oper Res.

[11] Falasca M, Zobel C, Cook D (2008). A decision support framework to assess supply chain resilience.

[12] Dun & Bradstreet Business Impact of the Coronavirus: Business and Supply Chain Analysis Due to the Coronavirus Outbreak.

[13] Gunasekaran A, Ngai E (2004). Information systems in supply chain integration and management. Eur J Oper Res.

[14] Kim SW, Narasimhan R (2002). Information system utilization in supply chain integration efforts. Int J Prod Res.

[15] Gonul Kochan C, Nowicki DR, Sauser B, Randall WS (2018). Impact of cloud-based information sharing on hospital supply chain performance: a system dynamics framework. Int J Prod Econ.

[16] Wang W, Chen L, Zhang Q (2015). Outsourcing high-dimensional healthcare data to cloud with personalized privacy preservation. Comput Netw.

[17] Barratt M (2004). Understanding the meaning of collaboration in the supply chain. Supply Chain Manag.

[18] Khuntia J, Ning X, Stacey R (2021). Digital orientation of health systems in the post-COVID-19 "new normal" in the United States: cross-sectional survey. J Med Internet Res.

[19] Kampstra R, Ashayeri J, Gattorna J (2006). Realities of supply chain collaboration. Int J Logist Manag.

[20] Cao M, Vonderembse MA, Zhang Q, Ragu-Nathan T (2009). Supply chain collaboration: conceptualisation and instrument development. Int J Prod Res.

[21] Gaynor M, Tuttle-Newhall J, Parker J, Patel A, Tang C (2020). Adoption of blockchain in health care. J Med Internet Res.

[22] Khurshid A (2020). Applying blockchain technology to address the crisis of trust during the COVID-19 pandemic. JMIR Med Inform.

[23] Cichosz M, Suchanek M (2021). Logistics startups and logistics service providers: competitors or partners in exploration?. Transport Development Challenges in the 21st Century. TranSopot 2019. Springer Proceedings in Business and Economics.

[24] Ishii L, Demski R, Ken Lee KH, Mustafa Z, Frank S, Wolisnky JP, Cohen D, Khanna J, Ammerman J, Khanuja HS, Unger AS, Gould L, Wachter PA, Stearns L, Werthman R, Pronovost P (2017). Improving healthcare value through clinical community and supply chain collaboration. Healthc (Amst).

[25] Langabeer J, Helton J (2021). Health Care Operations Management. 3rd ed.

[26] Hut N Reimagining the Healthcare Supply Chain to Bolster Resilience and Efficiency.

[27] Infosys BPM How Healthcare Supply Chains Can Move towards post-COVID Resilience.

[28] Motiwala SS, McLaughlin JE, King J, Hodgson B, Hamilton M (2008). Advancing the health care supply chain and promoting leadership through strategic partnerships with industry. Healthc Manag Forum.

[29] Nandi S, Sarkis J, Hervani AA, Helms MM (2021). Redesigning supply chains using blockchain-enabled circular economy and COVID-19 experiences. Sustain Prod Consum.

[30] Khuntia J, Ning X, Shekarian N, Stacey R (2021). Inaugural Health Systems’ Climate Study 2021.

